# Economic Evaluation of Enhanced Cleaning and Disinfection of Shared Medical Equipment

**DOI:** 10.1001/jamanetworkopen.2025.8565

**Published:** 2025-04-10

**Authors:** David Brain, Nirmali Sivapragasam, Katrina Browne, Nicole M. White, Philip L. Russo, Allen C. Cheng, Andrew J. Stewardson, Georgia Matterson, Peta E. Tehan, Kirsty Graham, Maham Amin, Martin Kiernan, Jennie King, Brett G. Mitchell

**Affiliations:** 1 Australian Centre for Health Services Innovation, School of Public Health and Social Work, Queensland University of Technology, Brisbane, Queensland, Australia; 2 School of Nursing and Health, Avondale University, Wahroonga, New South Wales, Australia; 3 Nursing and Midwifery, Monash University, Melbourne, Victoria, Australia; 4 Cabrini Health, Malvern, Victoria, Australia; 5 Infectious Diseases, Monash Health and School of Clinical Sciences, Monash University, Clayton, Victoria, Australia; 6 Department of Infectious Diseases, The Alfred and School of Translational Medicine, Monash University, Melbourne, Victoria, Australia; 7 School of Clinical Sciences, Faculty of Medicine, Nursing and Health Sciences, Monash University, Melbourne, Victoria, Australia; 8 Central Coast Local Health District, Gosford, New South Wales, Australia; 9 Richard Wells Research Centre, University of West London, Richard Wells Research Centre, The University of West London, Paragon House, Brentford, United Kingdom; 10 School of Nursing and Midwifery, University of Newcastle, Newcastle, New South Wales, Australia; 11 Hunter Medical Research Institute, Newcastle, New South Wales, Australia

## Abstract

**Importance:**

The economic value of cleaning and disinfection of shared medical equipment is currently unknown.

**Objective:**

To evaluate whether or not better environmental cleaning and disinfection of shared medical equipment would be cost-effective compared with usual care.

**Design, Setting, and Participants:**

This economic evaluation study was a within-trial cost-effectiveness analysis of a stepped-wedge cluster randomized clinical trial: the Cleaning and Enhanced Disinfection (CLEEN) study. The trial included 5002 inpatients and was conducted in 10 adult acute-care wards at a tertiary hospital in Australia between March 20, 2023, and November 24, 2023. The evaluation takes a hospital costing perspective. A decision-tree model was developed to evaluate the intervention; costs are presented in Australian dollars. Statistical analysis was performed from May to October 2024.

**Interventions:**

A multimodal cleaning intervention was introduced during the intervention, focusing on additional cleaning hours, education, audit, and feedback. The control group received usual care, including routine cleaning by clinical staff.

**Main Outcomes and Measures:**

Incremental cost-effectiveness ratio, where the mean change to costs associated with the intervention is divided by the mean change in outcomes.

**Results:**

This study assessed 5002 patients (2478 [49.5%] male, 2524 [50.5%] female [50.5%]; mean [SD] age, 71.6 [16.1] years). For a cohort of 1000 patients at risk of health care–associated infection (HAI), the estimated total costs associated with the intervention were $1 513 300, compared with $2 155 310 for usual care. The estimated number of HAIs was 100 in the intervention group, compared with 130 for the usual care group. Compared with usual care, the intervention was associated with reduced HAIs and costs, with a 90.5% chance that intervention adoption was cost-saving. This probability increased to 99.9% if a decision-maker was willing to pay $20 000 to avoid an infection.

**Conclusions and Relevance:**

In this economic evaluation study of enhanced cleaning and disinfection of shared medical equipment, the intervention resulted in reduced HAIs and a $642 010 reduction in costs per 1000 patients, compared with the control group. These results suggest that the CLEEN intervention is a cost-saving initiative.

## Key Points

**Question:**

Is enhanced cleaning and disinfection of shared medical equipment cost-effective compared with usual care?

**Findings:**

In this economic evaluation of a stepped-wedge cluster randomized clinical trial of 5002 patients, the cleaning intervention was associated with cost savings related to reduced health care-associated infections.

**Meaning:**

These findings suggest that investing in enhanced cleaning and disinfection of shared medical equipment could save hospitals money and improve patient safety.

## Teaser

This economic evaluation examines whether improved environmental cleaning and disinfection of shared medical equipment would be cost-effective compared with usual care.

## Introduction

Infection prevention remains a cornerstone of patient safety, requiring evidence-based strategies to guide clinical practices. One in 10 adult inpatients in Australian public hospitals acquire a health care–associated infection (HAI)^1^ resulting in more than 165 000 cases in Australia annually and contributes to approximately 7500 deaths each year.^2^ Despite the increased morbidity and mortality risks associated with HAIs, robust evidence supporting cost-effective infection prevention interventions is limited.

Environmental cleaning plays a critical role in infection prevention programs, albeit sometimes overlooked. Pathogens can survive on clinical surfaces for extended periods, creating reservoirs that facilitate transmission to patients.^3^ The nexus between environmental contamination and pathogen transmission in health care facilities is now well established,^4,5,6^ emphasizing the need for effective cleaning strategies.

The Researching Effective Approaches to Cleaning in Hospitals (REACH) trial demonstrated the cost-effectiveness of an environmental cleaning bundle focused on patient rooms.^7^ However, this study did not focus on the cleaning of shared medical equipment, which are used by multiple patients and should be decontaminated between use. Building on this foundation, the recent Cleaning and Enhanced Disinfection (CLEEN) study, conducted in 2023, was the first cluster randomized trial to assess the effect of improved cleaning and disinfection of shared medical equipment on HAI point prevalence.^8^ The CLEEN study evaluated a multimodal cleaning bundle that included additional dedicated cleaning hours, ongoing education, audit, and feedback mechanisms. The bundle was trialed in 10 wards of an Australian hospital using a cluster randomized, stepped-wedge design over 36 weeks. The results indicated that enhanced cleaning and disinfection protocols significantly reduced HAIs, underlining the importance for infection prevention.^9^ Moreover, the study highlighted the role of staff training, auditing, and feedback in maintaining high standards of cleanliness, thereby contributing to the overall success of infection prevention strategies in health care settings.

We conducted a within-trial cost-effectiveness analysis of the CLEEN study intervention, designed to reduce HAIs. Our aim is to provide health care decision-makers with robust evidence from an economic perspective. There is a paucity of cost-effectiveness evaluations for environmental cleaning interventions. Economic evaluations allow the clinical benefits and cost resources necessary to undertake treatments to be quantified—providing evidence supporting decisions about funding, reimbursement and investment in clinical services. Such evidence is of great value as health care decision-makers are tasked with balancing increased demand for services with a finite budget, in a time when hospital safety and quality is constantly in the spotlight.

## Methods

This economic evaluation follows the Consolidated Health Economic Evaluation Reporting Standards (CHEERS) checklist.^10^ The underpinning clinical trial received ethical approval from the Hunter New England Human Research Ethics Committee including a waiver of individual patient consent. Site-specific approval was granted by the participating hospital. The completed trial is registered with Australia New Zealand Clinical Trials Registry (ACTRN12622001143718).

### Health Economic Analysis Plan

A health economic analysis plan was developed alongside the clinical trial protocol (Supplement 1). The evaluation estimated the incremental cost-effectiveness ratio (ICER) of the CLEEN intervention vs usual care. ICERs were calculated by dividing the change in costs by the change in outcomes (HAIs). The ICER is compared against an a priori threshold, which is assumed to be the willingness to pay (WTP) for an increase in infections avoided. All ICERs that fall under this threshold are deemed to be cost-effective. Given that there is no reported threshold for infections avoided, we present our results using cost-effectiveness acceptability curves (CEACs), which show results according to thresholds ranging from $0 to $50 000 AUD per infection avoided. This approach provides decision-makers with differing budgets and opinions about the value of an avoided infection and allows them to decide about adoption based on their local conditions.

### Study Population, Setting, and Location

The protocol and statistical analysis plan for the stepped-wedge, cluster randomized trial that underpins this economic evaluation has been published,^8,11^ as have the clinical trial’s results.^9^ Ten wards from a large, public tertiary hospital in New South Wales, Australia, were recruited. To be eligible, wards within the hospital had to have more than 20 beds and care for adult patients. The included wards had a small proportion of single rooms, with most patients in 2 or 4-bed rooms.

### Comparators

Due to the stepped-wedge study design, all wards were exposed to the intervention and acted as their own control. In the trial’s control phase, cleaning staff were not responsible for cleaning shared medical equipment. The cleaning of shared medical equipment was the responsibility of clinical staff. The cleaning of shared medical equipment was very low during the control phase, increasing during the intervention.^9^ The multimodal intervention included an additional 3 hours of daily cleaning by dedicated cleaning staff (ie, there was no overlap between staff working on control and intervention wards). Extra cleaning as part of the intervention focused on shared medical equipment such as wheelchairs, blood pressure monitors, infusion pumps, and commodes. Products used to clean equipment did not change between the control and intervention. Combined detergent-disinfectant wipes registered with Australia’s Therapeutic Goods Administration were used on all pieces of equipment. Environmental cleaning audits were conducted every 2 weeks during the study to assess the thoroughness of cleaning. Education and training sessions for the intervention cleaning team were provided with an initial 1-hour session prior to the intervention, with refresher training repeated every 12-weeks or when auditing results showed cleaning thoroughness was below 50%. Full details on the intervention have previously been published.^9^

### Perspective, Time Horizon, and Discounting

This evaluation takes a hospital costing perspective, meaning that only those costs incurred in the hospital setting are included in the analysis. These include staff time, education, training, material development, consumables and/or equipment, treatment, medications and patient length of stay (LOS). Patient out-of-pocket costs and downstream health care costs beyond the hospital setting, such as readmission and lost productivity, were not included in this analysis, because they were not feasible to evaluate within the study design and timeframe. The model’s time horizon reflects the length of the trial (36 weeks) and as per guidelines for this kind of evaluation, does not include discounting of costs or health outcomes, due to the short time horizon.

### Outcome Selection, Measurement, and Valuation

The outcome of interest was confirmed cases of HAI, as defined in the European Centre for Disease Prevention and Control (ECDC) protocol, version 5.3.^12^ These included but were not limited to pneumonia, urinary tract, surgical site, and bloodstream infections. HAIs were defined as any infection acquired as a direct or indirect result of health care more than 48 hours after admission or procedure. To identify active HAIs, point prevalence surveys were undertaken every 2 weeks, with data collected for all adult patients admitted to a study ward. Following a review of medical, pathology, and microbiology records, the determination of a HAI was undertaken using an algorithm applying the ECDC HAI definitions.^8,9^ The data collector was blinded to the intervention allocation.

### Measurement and Valuation of Resources and Costs

Costing information was collected during the trial and followed a previously published method for costing infection control interventions.^13^ Costs associated with LOS attributable to infection were derived from the literature^14,15,16^ (Table 1). All estimates of excess LOS for infections were based on multistate models which considered the time-dependent nature of infections.^17^ An inventory of consumable use was kept by the trial coordinator, with total use multiplied by product cost to ascertain estimates for the model. Staff time, including that required for training, extra cleaning, audit, and feedback were recorded via timesheets with local health service pay scales used to calculate staff costs. All other data used to furnish the model was estimated from the clinical trial. All transition probabilities—the likelihood of moving from one of the tree’s branches to another—were derived from the CLEEN study, for both the intervention and usual care. Costs associated with each branch of the decision-tree were derived from the trial and included staff time, trainer time, audit, and feedback costs, consumables and product (cleaning wipes, ultraviolet torches) required to carry out the intervention.

**Table 1.** Input Variables for the Decision-Analytic Model

Variable	Distribution	Mean (SD)	Source/comments
Transition probabilities^a^			
HAI (usual care)	Beta	0.13 (<0.01)	Trial data
BSI (usual care)	Beta	0.09 (<0.01)	Trial data
PN (usual care)	Beta	0.07 (0.01)	Trial data
SSI (usual care)	Beta	0.17 (<0.01)	Trial data
UTI (usual care)	Beta	0.13 (0.02)	Trial data
GI (usual care)	Beta	0.14 (<0.01)	Trial data
CDI (usual care)	Beta	0.05 (0.01)	Trial data
LRI (usual care)	Beta	0.04 (0.01)	Trial data
EENT (usual care)	Beta	0.15 (0.02)	Trial data
Other infection (usual care)^b^	Beta	0.17 (<0.01)	Trial data
HAI (intervention)	Beta	0.10 (<0.01)	Trial data
BSI (intervention)	Beta	0.04 (<0.01)	Trial data
PN (intervention)	Beta	0.11 (0.02)	Trial data
SSI (intervention)	Beta	0.17 (<0.01)	Trial data
UTI (intervention)	Beta	0.16 (0.02)	Trial data
GI (intervention)	Beta	0.17 (<0.01)	Trial data
CDI (intervention)	Beta	0.05 (0.01)	Trial data
LRI (intervention)	Beta	0.01 (<0.01)	Trial data
EENT (intervention)	Beta	0.22 (0.02)	Trial data
Other infection (intervention)^b^	Beta	0.08 (<0.01)	Trial data
Excess LOS			
BSI	Normal	11.4 (2.8)	Stewart et al,^14^ 2021
PN	Normal	16.3 (4.5)	Stewart et al,^14^ 2021
SSI	Normal	9.8 (2.7)	Stewart et al,^14^ 2021
UTI	Normal	4.0 (0.5)	Mitchell et al,^16^ 2016
GI	Normal	6.0 (3.4)	Stewart et al,^14^ 2021
CDI	Normal	0.9 (3.7)	Mitchell et al,^15^ 2014
LRI	Normal	7.3 (2.8)	Stewart et al,^14^ 2021
EENT	Normal	0	Expert opinion
Other infection^b^	Normal	14 (9.1)	Stewart et al,^14^ 2021
Costs, $AUD			
Audit and feedback time	Fixed	3537	Trial data
Staff training time	Fixed	2358	Trial data
Trainer time	Fixed	472	Trial data
Staffing of additional cleaning shifts	Fixed	106 110	Trial data
Sporicidal wipes	Fixed	1134	Trial data
Universal wipes	Fixed	9737	Trial data
Biodegradable wipes	Fixed	15 288	Product quote
Indicator tags	Fixed	1318	Trial data
Pens	Fixed	99	Trial data
Auditing torch and markers	Fixed	116	Trial data
Intervention cost (per patient)	Gamma	50.07 (50.07)	Trial data
Daily LOS cost	Gamma	2151 (2151)	IHACPA

Abbreviations: BSI, bloodstream infection; CDI, *Clostridioides difficile* infection; EENT, ear, eyes, nose, mouth, and throat infection; GI, gastrointestinal infection; HAI, health care-associated infection; IHACPA, Independent Health and Aged Care Pricing Authority; LOS, length of stay; LRI, lower respiratory infection; PN, pneumonia; SSI, surgical site infection; UTI, urinary tract infection.

^a^ Probability of having the infection.

^b^ Other infections included cardiovascular system infection; ears, eyes, nose, throat or mouth infection; skin or soft tissue, or systematic infection.

### Statistical Analysis

#### Currency, Price Date, and Conversion

The evaluation was conducted in 2023 Australian dollars, which is in line with the year the trial was conducted. Due to the recency of the trial being completed compared with this analysis being undertaken, no currency conversion was required. At the time of writing, 1 AUD was approximately equal to 0.65 USD or 0.62 EUR.^18^

#### Rationale, Model Description, and Assumptions

**Figure 1.**
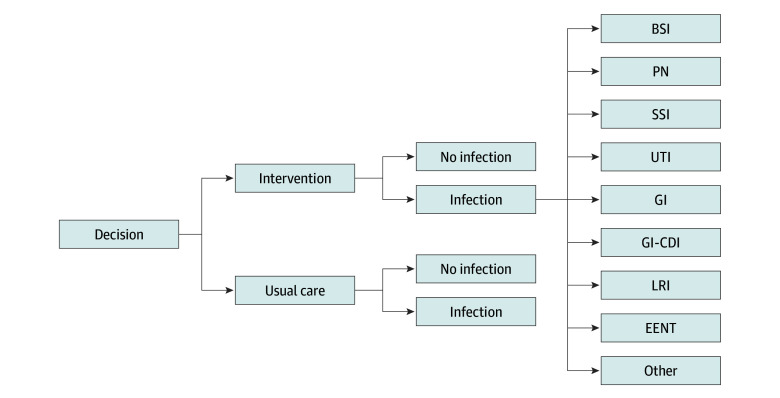
Decision-Tree for Possible Paths of Movement for Patients The usual care branches of the tree are the same as the intervention group (ie, include the same infections). They are omitted from this decision-tree to help reduce complexity and aid visual interpretation. BSI indicates bloodstream infection; EENT, ear, eye, nose, mouth, and throat infection; GI, gastrointestinal infection; GI-CDI, gastrointestinal infection – *Clostridioides difficile*; LRI, lower respiratory infection; PN, pneumonia; SSI, surgical site infection; UTI, urinary tract infection. Other included cardiovascular system infection, EENT, skin or soft tissue, and systematic infection.

A decision-tree model was developed to evaluate the intervention, where branches of the tree represent possible outcomes for the cohort of patients who move through the model. A pictorial representation of the model shows the possible paths of movement for patients and is shown in Figure 1. Due to the way clinical data were recorded, the model assumed that only 1 infection was possible per cycle. This is a simplification of the real world and likely represents an underestimation of the extra costs associated with having a health care–associated infection. Each of the model’s end points had a cost ($AUD) and health outcome (HAIs) associated with it. The probability of moving around the model was informed by data collected from the clinical trial. The model was constructed and analyzed in TreeAge Pro (version 2022 R2.0) and cross-referenced in Microsoft Excel.

#### Characterizing Uncertainty

Probabilistic sensitivity analysis was undertaken to measure the effect of uncertainty relating to the model’s inputs on cost-effectiveness results. One thousand iterations of the model were run using Monte Carlo simulation, with each simulation including random draws from each parameter’s distribution. Each simulation produces an individual ICER, which when all iterations of the model are complete, are plotted on a cost-effectiveness scatter plot to show the distribution of expected outcomes.

To further explore the effect of uncertainty on results and test the model’s robustness, we undertook scenario analyses. This process requires key parameter values to be changed, reflecting plausible decision-making scenarios beyond the setting of the clinical trial. Two alternate scenarios were examined. In scenario 1, we explored the impact of using more expensive biodegradable wipes which contain identical disinfectants as those used in the trial. This scenario was chosen to provide decision-makers with economic evidence that incorporates environmental sustainability considerations. In scenario 2, we explored the impact of a lower effectiveness outcome, by halving the intervention’s effectiveness estimate. The effect size found in the CLEEN study was considerably high, hence we wanted to explore the cost-effectiveness of the intervention for hospitals that may not see the same effect. Statistical analysis was performed from May to October 2024

## Results

The primary analysis had a total sample size of 5002 adult patients (2478 [49.5%] male, 2524 [50.5%] female; mean [SD] age, 71.6 [16.1] years). Patient characteristics are published in full elsewhere.^9^ For ease of reporting and lay-interpretation, we have reported the population as a 1000-patient cohort, using the trial-derived probabilities so the model reflects what occurred within the trial.

The comparable probabilities of infection, associated LOS and costs to implement the study are presented in Table 1. The probability distribution of HAIs given an infection ranged from 0.01 (LRI, usual care group) to 0.22 (EENT, intervention group). Compared with the control, there was a reduction in the number of HAIs in the intervention.

### Analysis of Main Results

For a cohort of 1000 people, the estimated total costs associated with the intervention were $1 513 300, compared with $2 155 310 for usual care (Table 2). The estimated number of HAIs was 100 in the intervention group, compared with 130 for the usual care group. Due to the intervention being less costly and more effective, it dominated usual care. The intervention also affected LOS and accounted for a reduction in bed-day occupation. Total excess bed-days for the intervention group was 922, whereas the total excess bed-days for the control group was 1306, resulting in the intervention saving 384 excess bed-days.

**Table 2.** Cost-Effectiveness Analysis for 1000 Patients

Group	Total costs, $AUD^a^	Total HAIs	Change in costs, $AUD	HAIs avoided	ICER
Usual care	2 155 310	130	NA	NA	NA
Intervention	1 513 000	100	−642 010	30	Dominant^b^

Abbreviations: ICER, incremental cost-effectiveness ratio; NA, not applicable.

^a^ Total costs refer to staff time, education, training, material development, consumables and/or equipment, treatment, medications, and patient length of stay. In usual care, the costs were related to length of stay.

^b^ Due to the intervention’s reduction in costs and HAIs, usual care is said to be dominated by the intervention.

### Effect of Uncertainty

**Figure 2.**
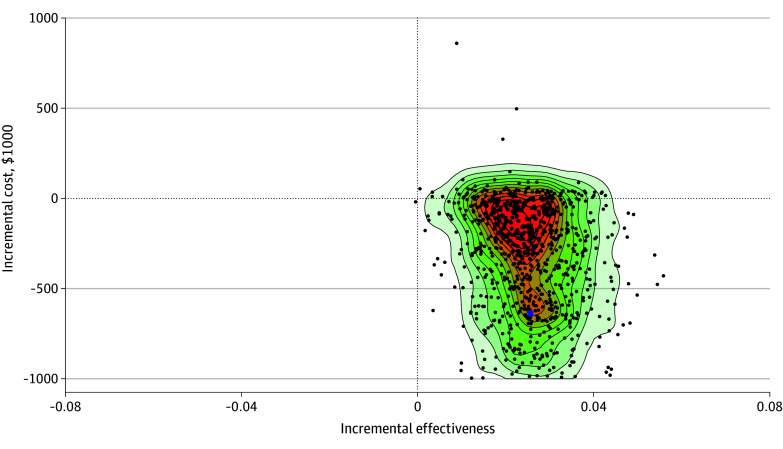
Heatmap Showing Distribution of Incremental Cost-Effectiveness Ratios From the Probabilistic Sensitivity Analysis The blue square is the mean of the incremental cost-effectiveness ratios from the probabilistic sensitivity analysis.

The results of the Monte Carlo simulation can be seen in Figure 2, which shows the distribution of ICERs across the cost-effectiveness plane. There is a concentration of ICERs shown in the red core of the figure, with the majority (90.5%) of all ICERs falling in the cost-saving quadrant (bottom right) of the plane.

Given that there is not an agreed willingness-to-pay threshold for an avoided infection, we also produced cost-effectiveness acceptability curves, showing the probability of the intervention being cost-effective at different thresholds. Thresholds ranged from $0 per infection avoided to $50 000 per infection avoided, in $5000 increments (Table 1). The intervention has a 90.5% probability of being cost-effective when the threshold is at its most severe: $0 per infection avoided. This is akin to saying that there is a 90.5% chance the intervention will be cost saving. This probability increased to 99.9% if a decision-maker was willing to pay $20 000 to avoid an infection, with the probability reaching 100% when the willingness-to-pay threshold is $30 000 per infection avoided, or higher. As seen in Figure 2, at no point do the curves cross, meaning that there is no scenario where usual care is deemed to be better value for money in comparison with the intervention.

### Scenario Analyses

Two scenarios were examined, testing the model’s robustness and providing decision-makers with a wider breadth of new information. Scenario 1 involved more expensive biodegradable wipes. Scenario 2 represented a halving of the effectiveness estimate. Table 3 shows the results of the sensitivity analyses.

**Table 3.** Cost-Effectiveness Results From Scenario Analysis for 1000 Patients

Group	Mean (95% CI)				ICER
	Total costs, $AUD	Total HAIs	Change in costs, $AUD	HAIs avoided	
**Scenario 1**					
Usual care	2 149 214 (2 008 458 to 2 289 971)	129.43 (129.02 to 129.84)	NA	NA	NA
Intervention	1 512 080 (1 417 140 to 1 607 019)	103.70 (103.33 to 104.08)	−637 135 (−690 439 to −583 830)	25.73 (25.17 to 26.28)	Dominant^a^
**Scenario 2**					
Usual care	2 149 214 (2 008 287 to 2 290 142)	129.43 (129.02 to 129.84)	NA	NA	NA
Intervention	1 689 408 (1 582 688 to 1 796 128)	116.45 (116.06 to 116.85)	−459 806 (−504 442 to −415 170)	12.98 (12.41 to 13.54)	Dominant^a^

Abbreviations: HAI, health care-associated infections; ICER, incremental cost-effectiveness ratio; NA, not applicable.

^a^ Due to the intervention’s reduction in costs and HAIs, usual care is said to be dominated by the intervention.

Even when more expensive consumables were used for the intervention, it was dominant in comparison with usual care (scenario 1) (Table 3). The use of biodegradable wipes, impregnated with the same disinfectant as those used in the trial, were associated with increased consumable costs but had no negative consequences from a value-for-money perspective. eFigure 2 in Supplement 1 shows the cost-effectiveness acceptability curves for this scenario analysis.

Despite halving the effectiveness of the intervention, it remained good value for money across a range of willingness-to-pay thresholds. Even at a threshold of $0 per infection avoided, the intervention was cost-saving in 83% of all simulations. As the cost-effectiveness threshold increased, so did the probability of the intervention being cost-effective—at a threshold of $20 000 per infection avoided, the intervention was cost-effective in 94% of simulations—and increased to 95% probability of being cost-effective at a threshold of $50 000 per infection avoided. eFigure 3 in Supplement 1 shows the cost-effectiveness acceptability curves for this scenario analysis.

## Discussion

In our cost-effectiveness evaluation, we found that the enhanced cleaning and disinfection of shared medical equipment was both less expensive and more effective at preventing HAIs than usual care. Per 1000 hospitalized patients, the intervention reduced 30 HAIs and released 384 hospital bed days otherwise occupied due to HAI-related care. In this study, the model’s results were resistant to changes in key parameters in our scenario analyses. The relative HAI reduction of 34.5% in the CLEEN study may be considered high by some. However, when we modeled halving the effectiveness of the intervention, the intervention remained cost saving. The evidence we present in our analysis demonstrates that investment in a cleaning and disinfection of shared medical equipment improves patient safety and overall hospital efficiency. If implemented across Australian public hospitals, we estimate 1900 HAIs could be prevented and over 24 000 hospital bed days released per year (Supplement 1). If implemented worldwide, we estimate that these cost savings would be substantially higher.

In clinical practice, the clean-between-use model often relies on clinical staff for cleaning, but compliance is low, as shown by CLEEN and other studies.^19^ The CLEEN study demonstrated what others have also reported: that such cleaning rarely occurs.^20,21,22,23^ Cleaning of shared medical equipment takes training and considerable time, as evidenced by a recent time and motion study.^24^ While clinical staff could be trained and allocate more time for cleaning, their higher costs may make this approach less sustainable than using cleaning staff. Future work will explore alternative delivery models for shared equipment cleaning to provide options tailored to hospital needs.

Transparent cost-effectiveness evaluations help inform evidence-based infection control guidelines, a point well made in a commentary by Gould and Drey in response to the CLEEN study.^25^ Few RCTs have assessed environmental cleaning. We are aware of only 1 other cost-effectiveness evaluation of a RCT involving environmental cleaning, the REACH study.^7^ White and colleagues^7^ found that introducing an environmental cleaning bundle for routine and terminal cleaning in hospitals was cost-effective. Combined with our findings, these data support structured investment in environmental cleaning to improve patient outcomes and hospital efficacy.

Preventing HAIs has broader implications beyond reducing patient morbidity and mortality. HAIs prolong hospital stays, increasing resource use and limiting bed availability, contributing to issues such as overcrowding and ambulance ramping, thus there is a cost associated with failing to prevent HAIs.^14^

In the delivery of health services, there are large volumes of clinical waste. Sustainability is vital, but should not compromise provision of safe, quality care. We must always seek to use the strongest evidence base for health care practice. To inform a decision around a more sustainable approach to our intervention, we changed our input from plastic to a more expensive, biodegradable wipe which contains the same disinfectant. Despite the higher purchase cost of biodegradable wipes, the intervention remained cost-saving, underscoring the economic feasibility of integrating sustainability into infection prevention strategies. This finding reflects the relatively low proportion of consumable costs within the overall intervention budget. In the context of these 2 scenarios, decision-makers who invest can have confidence that their investment is sound.

### Limitations

This study has limitations. This conservative analysis may underestimate savings, as we only accounted for a single HAI per patient, though 124 CLEEN study participants had multiple HAIs. Additional LOS due to HAIs was derived from studies that adjusted for time-dependent bias, ensuring conservative estimates but potentially overlooking variations between infection types (eg, superficial vs deep surgical site infections). For EENT infections, a lack of reliable data led us to assign 0 additional LOS, further affirming the conservative nature of our approach. We did not include quality-adjusted life years in our analysis, focusing instead on a hospital perspective to align with the cleaning funders’ priorities. While reducing HAIs has societal benefits, hospitals, as the primary funders of cleaning, do not directly benefit from extended life expectancy. Additionally, our stepped-wedge cluster trial design, while robust, was not powered for individual infections, and transition probabilities did not account for clustering. Additionally, our evaluation used data from a single site, which may not reflect the diverse clinical landscape. However, our study addressed a universal problem and provides an implementation model that was cost-effective in this study’s simulations.

## Conclusions

In this economic evaluation study of enhanced cleaning and disinfection of shared medical equipment, the intervention resulted in reduced HAIs and a $642 010 reduction in costs, per 1000 patients, compared with the control group. In an era of stagnant budgets, decision-makers should be looking to maximize health gain per dollar spent and make sensible economic decisions. This study’s results suggest that the CLEEN intervention is a cost-saving initiative and may provide an opportunity to maximize health gain from a scarce budget.

## Notes

Supplement 1. **eAppendix 1. **Health Economic Analysis Plan **eFigure 1.** Cost-Effectiveness Acceptability Curve Comparing Probability of Intervention and Usual Care Being Cost-Effective **eFigure 2.** Cost-Effectiveness Acceptability Curve for Scenario Analysis 1 (Use of Biodegradable Wipes) **eFigure 3.** Cost-Effectiveness Acceptability Curve for Scenario Analysis 2 (Halved Effectiveness) **eAppendix 2.** CLEEN Study Translation to Australian Public Hospitals

Supplement 2.
Data Sharing Statement
