# Supplementary material for: Economic Evaluation of Enhanced Cleaning and Disinfection of Shared Medical Equipment
Source: JAMA Netw Open. 2025 Apr 10;8(4):e258565. doi: 10.1001/jamanetworkopen.2025.8565 (PMC11986775; doi:10.1001/jamanetworkopen.2025.8565)
Supplement: Supplement 1. — eAppendix 1. Health Economic Analysis Plan eFigure 1. Cost-Effectiveness Acceptability Curve Comparing Probability of Intervention and Usual Care Being Cost-Effective eFigure 2. Cost-Effectiveness Acceptability Curve for Scenario Analysis 1 (Use of Biodegradable Wipes) eFigure 3. Cost-Effectiveness Acceptability Curve for Scenario Analysis 2 (Halved Effectiveness) eAppendix 2. CLEEN Study Translation to Australian Public Hospitals [file jamanetwopen-e258565-s001.pdf]

Supplemental Online Content

Brain D, Sivapragasam N, Browne K, et al. Economic evaluation of enhanced cleaning and disinfection of shared medical equipment. *JAMA Netw Open.* 2025;8(4):e258565. doi:10.1001/jamanetworkopen.2025.8565

eAppendix 1. Health Economic Analysis Plan

eFigure 1. Cost-Effectiveness Acceptability Curve Comparing Probability of Intervention and Usual Care Being Cost-Effective

eFigure 2. Cost-Effectiveness Acceptability Curve for Scenario Analysis 1 (Use of Biodegradable Wipes)

eFigure 3. Cost-Effectiveness Acceptability Curve for Scenario Analysis 2 (Halved Effectiveness)

eAppendix 2. CLEEN Study Translation to Australian Public Hospitals

This supplemental material has been provided by the authors to give readers additional information about their work.

© 2025 Brain D et al. *JAMA Network Open*.

eAppendix 1. Health economic analysis plan

A within-trial cost-effectiveness analysis was planned from the outset of the study and included in the published trial protocol, where evaluating the intervention from an economic perspective was listed as a secondary outcome [1,2]. The cost-effectiveness analysis was planned by the entire research team, led by project CI Mitchell and project health economist, CI Brain.

Title: Economic evaluation of enhanced cleaning and disinfection of shared medical equipment (CLEEN)

Roles & Responsibilities: CI Brain (health economist) to lead the economic evaluation. CI White (statistician), CI Mitchell (project lead) and CI Browne to provide first-line support. The remaining CI and AI group to provide input, as needed. Meetings to progress the economic evaluation after the trial were planned, diarised and undertaken on an as-needed basis.

Objective: To determine whether enhanced cleaning and disinfection of shared medical equipment is cost-effective compared with usual care.

Background Rationale: In an era of non-increasing budgets, economic evidence is of growing importance to healthcare decision-makers and is largely required to support requests for hospital funding. Hospital-acquired infections are a known issue in all hospital settings and contribute to increased lengths of stay, morbidity and mortality. Infection control, including environmental cleaning interventions, are a practical and important way to intervene in transmission, keeping patients and staff safe. There is no economic evidence available relating to the cost-effectiveness of cleaning shared medical equipment in the Australian setting. This analysis will provide decision-makers with new and useful information that will help them make evidence-based funding allocation decisions.

Methods: The within-trial economic evaluation will take place under the intention to treat principle. A hospital costing perspective will be taken for the base-case analysis with all costs valued and reported in Australian dollars. The time horizon will match the trial length. No discounting of costs or health outcomes will be undertaken if the time horizon does not exceed 12 months. We will use a decision-analytic model, namely a decision-tree, to model the outcomes of the trial. Cost-effectiveness will be shown using the incremental cost-effectiveness ratio, where the mean change to costs associated with the intervention is divided by the mean change in health outcomes. Resource use, costs and health outcomes will be collected for all

© 2025 Brain D et al. *JAMA Network Open*.

participants enrolled in the trial and will be used to parameterise the model. Patient inclusion follows the plan outlined in the clinical trial protocol. For intervention-related costs such as staffing costs, cleaning product and consumables, we will collect and record appropriate data from the clinical trial. The evaluation's results will be reported according to the Consolidated Health Economic Evaluation Reporting Standards (CHEERS). The primary health outcome of this evaluation is healthcare-associated infections. We will account for individual patients having multiple infections at the same time when counting total infections, but to avoid double counting costs associated with additional length of stay we will only use the length of stay associated with the more severe infection. To account for additional length of stay due to HAIs we will only use literature that accounted for time dependent bias in their estimates. Where robust estimates for extra length of stay are not available, we will report additional length of stay as zero – a conservative approach. To account for uncertainty in model inputs, we will undertake probabilistic sensitivity analysis using Monte Carlo simulation, running the model 1,000 times. We will undertake two scenario analyses – first, to model the cost-effectiveness of using more expensive, biodegradable wipes that contain the same cleaning agent as those used in the trial. Given the intervention is from a single-site study, we will model different intervention effect sizes, to increase the likelihood that decision-makers have results that are closer to their local conditions.

Data: Data will be managed according to the specifications laid out in the clinical trial protocol, adhering to all confidentiality, ethics and governance approvals. Any data that is required for the model that is not from the trial itself will be sourced as per evidence-synthesis guidelines published elsewhere[3].

Analysis Plan: The decision analytic model will be programmed in TreeAge Pro and independently in MS Excel for comparison. The structure of the model will be decided upon by the entire research team which includes a health economist, the trial statistician, trial manager and numerous clinicians to ensure that it is a fair representation of reality. Changes in the primary outcome (HAIs) will be used to estimate changes in health benefits from cases prevented by the intervention. Change to costs associated with resource use, intervention costs and length of stay will be compared between the intervention and the control groups. Uncertainty in parameter estimates will be captured using appropriate statistical distributions to describe the variability (beta for transition probabilities; gamma or fixed for costs), the fitted distributions subject to random re-sampling via probabilistic sensitivity analysis. Cost-

© 2025 Brain D et al. *JAMA Network Open*.

effectiveness will be determined by the cost per infection avoided, with 1,000 iterations of the model plotted on a cost-effectiveness plane and cost-effectiveness acceptability curves, with threshold values between $0 and $50,000 per infection avoided.

© 2025 Brain D et al. *JAMA Network Open*.

Supplementary Figures

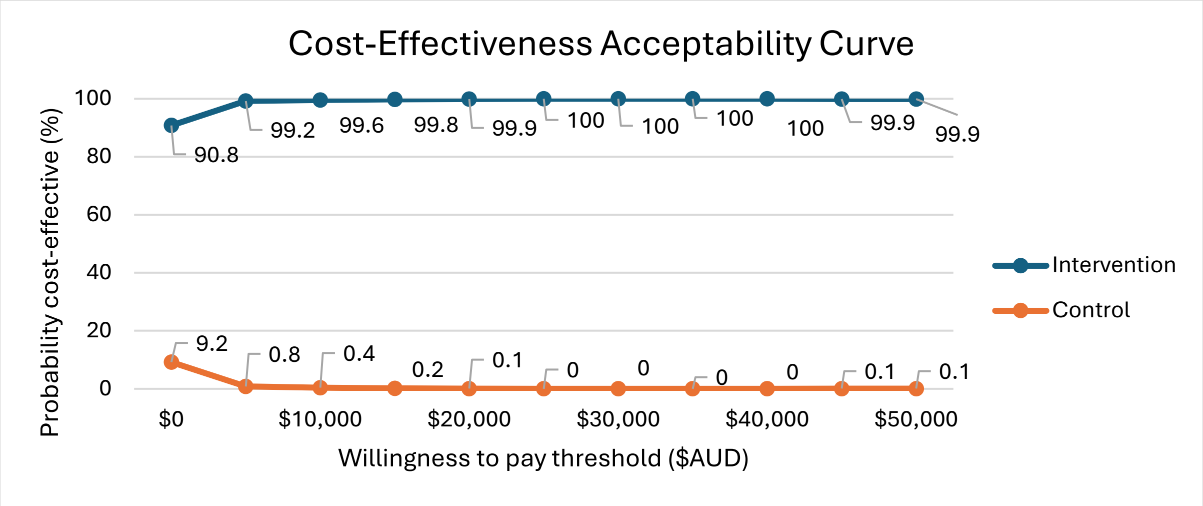

eFigure 1. Cost-effectiveness acceptability curve comparing probability of intervention and usual care being cost-effective.

Supplementary Figure 2

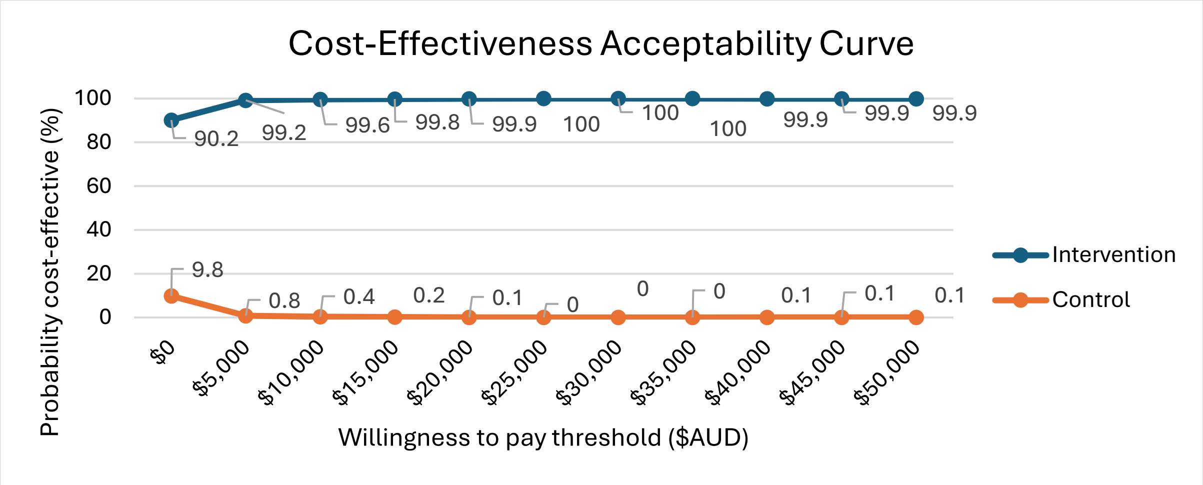

eFigure 2. Cost-effectiveness acceptability curve for scenario analysis 1 (use of biodegradable wipes).

Supplementary Figure 3

© 2025 Brain D et al. *JAMA Network Open*.

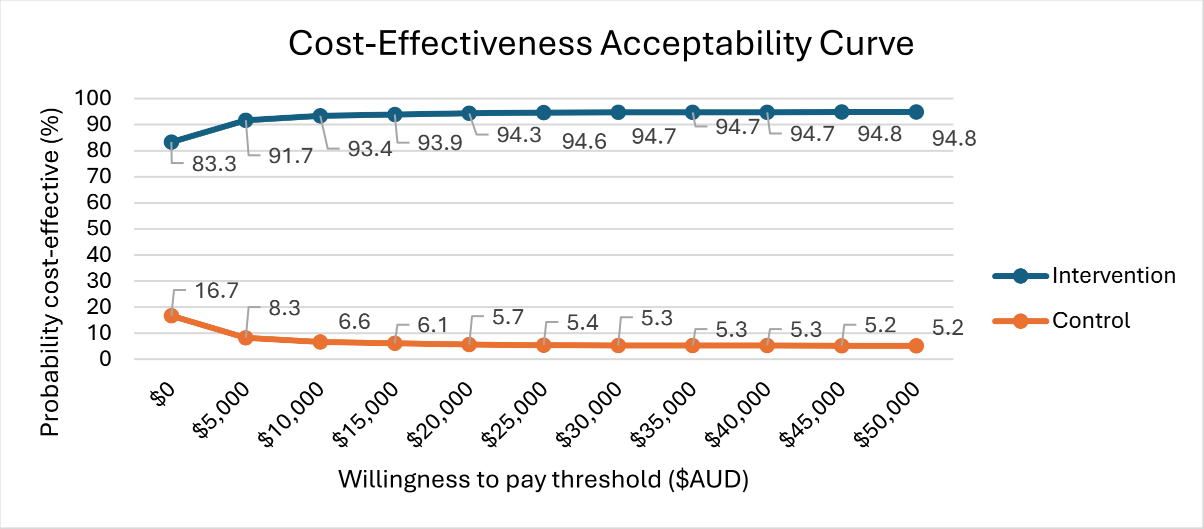

eFigure 3. Cost-effectiveness acceptability curve for scenario analysis 2 (halved effectiveness).

© 2025 Brain D et al. *JAMA Network Open*.

eAppendix 2. CLEEN Study Translation to Australian Public Hospitals

Assumptions

- The CLEEN study demonstrated a reduction of 30 HAIs and a release of 384 bed days per 1000 patients associated with the intervention.
- There are approximately 64,000 hospital beds in Australia (Australian Government, Australian Productivity Commission, Report of Government Services 2024).
- One additional staff member was employed for 3 hours per weekday, per ward, during the intervention.

Wards had a mix of single, double and quad rooms.

CLEEN study translation

If the CLEEN intervention was translated in full

- Potential HAI reduction across Australia: 64,000/1000 * 30 = 1,920 HAIs
- Potential bed days to be freed across Australia: 64,000/1000 * 383 = 24,512 bed days.

© 2025 Brain D et al. *JAMA Network Open*.

eReferences

1. Browne K, White NM, Russo PL, et al. Investigating the effect of enhanced cleaning and disinfection of shared medical equipment on health-care-associated infections in Australia (CLEEN): a stepped-wedge, cluster randomised, controlled trial. *Lancet Infect Dis*. Aug 13 2024;doi:10.1016/S1473-3099(24)00399-2

2. Browne K, White N, Tehan P, et al. A randomised controlled trial investigating the effect of improving the cleaning and disinfection of shared medical equipment on healthcare-associated infections: the CLEaning and Enhanced disiNfection (CLEEN) study. *Trials*. Feb 22 2023;24(1):133. doi:10.1186/s13063-023-07144-z
