# Supplementary material for: Economic Evaluation of Enhanced Cleaning and Disinfection of Shared Medical Equipment
Source: JAMA Netw Open. 2025 Apr 10;8(4):e258565. doi: 10.1001/jamanetworkopen.2025.8565 (PMC11986775; doi:10.1001/jamanetworkopen.2025.8565)
Supplement: Supplement 2. — Data Sharing Statement [file jamanetwopen-e258565-s002.pdf]

Data Sharing Statement

Brain. Economic Evaluation of Enhanced Cleaning and Disinfection of Shared Medical Equipment. *JAMA Netw Open*. Published April 10, 2025.
doi:10.1001/jamanetworkopen.2025.8565

Data
Data available: No

Additional Information
Explanation for why data not available: Data will be available upon request with relevant ethical approvals.
